# Poisoning after ingestion of pufferfish in Brazil: report of 11 cases

**DOI:** 10.1186/1678-9199-20-54

**Published:** 2014-12-09

**Authors:** Eneida Márcia de Souza Simões, Thelma Marly Abreu Mendes, Angelino Adão, Vidal Haddad Junior

**Affiliations:** Secretariat of Health of Duque de Caxias City, Caxias City, Rio de Janeiro State Brazil; Department of Dermatology, Botucatu Medical School, São Paulo State University (UNESP – Univ Estadual Paulista), Caixa Postal 557, Botucatu, SP CEP 19618-000 Brasil

**Keywords:** Pufferfish, Marine poisonings, Poisonous animals, Poisonous fish, Tetrodotoxin

## Abstract

In this brief communication the authors report eleven cases of human poisoning caused by ingestion of pufferfish meat. Three patients (two children and one adult) were seriously affected. The circumstances that precipitated the poisoning are discussed as well as the clinical aspects observed. No deaths were registered and the patients did not present sequelae after the episode.

## Dear Editor of *JVATiTD*

Pufferfish or blowfish (*baîaku*, in ancient Tupian language) are marine bony fish commonly found in tropical areas. They are capable of inflating their body by swallowing air or water, assuming a rounded shape that makes difficult the predation by other animals. Additionally, pufferfish may store toxins synthesized by bacteria that cause severe poisoning when its viscera, skin or muscles are ingested [1–5].

The majority of victims of pufferfish poisoning are fishermen and their families that usually consume their meat [1–5]. In Japan, the approximate number of poisonings per year is 50 with a high death rate [1–5]. In that country, there is a typical dish that employs raw pufferfish meat (*fugu*). In Brazil, the most important genera are *Lagocephalus* and *Sphoeroides*. The former accumulates less toxins and is not commonly associated with poisoning, while the latter is related to most accidents in the country [1–5].

The most common pufferfish toxin is the tetrodotoxin, a neurotoxin with violent paralyzing effect, which blocks sodium channels. The symptoms usually begin within six hours with perioral paresthesia, weakness of facial muscles and extremities, abdominal pain, drooling, nausea, vomiting, and diarrhea. Patients may have motor dysfunction with muscular weakness, hypoventilation, and dysarthria. The ascending paralysis occurs within 4 to 24 hours, with paralysis of the extremities followed by paralysis in respiratory muscles. Late cardiac and central nervous system dysfunction may be observed. Patients with severe poisoning may develop important muscular paralysis, have fixed and non-reactive pupils, apnea, and absence of encephalic trunk reflexes, but they remain aware. Victims who survive the acute phase of intoxication (the first 24 hours), usually recover without long term consequences, but full recuperation may take days. There is no antidote, and the treatment is based on responding to manifested signs and symptoms [1–5].

In this communication, we report the poisoning of 11 members of a family from Duque de Caxias city (Rio de Janeiro state), which occurred within anomalous circumstances and outside the profile of accidents observed in Brazil [1–5].

**Figure 1:**
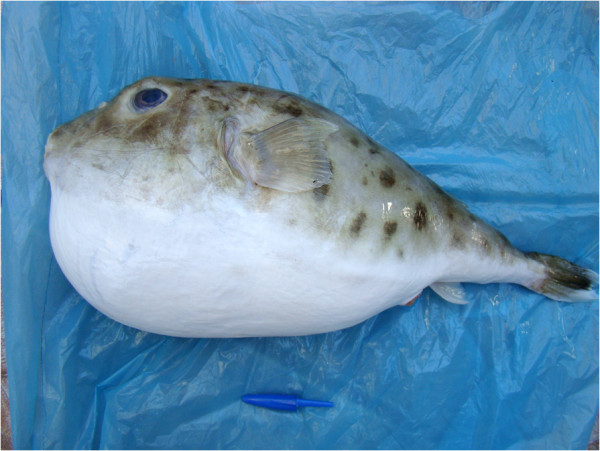
**A specimen of the genus** ***Sphoeroides*** **, the spotted puffer fish or** ***baiacu-pinima*** **(in Portuguese).** This genus is associated with most poisonings in Brazil.

The poisonings occurred after the consumption of fish fillets that included pufferfish meat at a lunch meeting with the family and their friends, in which 22 people were present. One of the residents got the frozen fillet of spotted pufferfish from her aunt (caught by the stepfather of the victim, who discarded the bigger pufferfish, but did not notice the smaller ones. It is believed that the fillets were prepared with these smaller fish. Generally, *Lagocephalus* pufferfish are bigger than those of the genus *Sphoeroides* (Figure 1). The fish fillets were gathered in one container, seasoned and fried together. Only the ones who ate pufferfish fillet got sick (11 people), with neuromuscular symptoms appearing 20 minutes after ingestion in the first patient and two hours after in the last.

Of the people who became ill, eight lived in the same house. According to the reports of the patients, only after the ingestion and onset of symptoms they realized that they had eaten pufferfish. The initial symptoms included paresthesia in the perioral region and nausea/vomiting. The patients were taken to various health centers of the municipality.

One child had discrete manifestations and was released after observation. Another ten patients developed moderate symptoms, such as mild muscular weakness, and were hospitalized for observation.

Three patients were severely affected (one adult and two children) and were treated in an intensive care center. The adult had a cardiac arrest and one child developed mydriatic pupils, but with response to light stimulation. Both were diagnosed with respiratory failure and received mechanical ventilatory support and therapeutic clinical support, which comprises a critical situation in this type of accident. All patients improved their health condition and were discharged, initially those with moderate symptoms and later the seriously ill. The last patient (a child that was in the intensive care unit) was discharged without symptoms after ten days.

The patients had no sequelae. This series of poisonings has some peculiarities: despite the severity of the condition caused by poisoning, no deaths occurred, although two patients developed severe symptoms. The therapeutic action was appropriate and epidemiological work has shown that the poisonings happened accidentally, as fishermen and residents of the region do not have the habit of consuming pufferfish. There is no need for awareness campaigns in that area, since their inhabitants usually avoid consuming pufferfish due to the risk of poisoning.

## Consent

Written informed consent was obtained from the victims for publication of this article.

## References

[1] Haddad V, Takehara ET, Rodrigues DS, Lastória JC (2004). Envenenamento por baiacus (peixes-bola): revisão sobre o tema. Diagn Tratamento.

[2] Haddad V, Lupi O, Lonza JP, Tyring SK (2009). Tropical dermatology: marine and aquatic dermatology. J Am Acad Dermatol.

[3] Silva CCP, Zannin M, Rodrigues DS, Santos CR, Correa IA, Haddad V (2010). Clinical and epidemiological study of 27 poisonings caused by ingesting puffer fish (Tetrodontidae) in the states of Santa Catarina and Bahia, Brazil. Rev Inst Med Trop Sao Paulo.

[4] Santana Neto PL, de Aquino EC M, Silva JA, Porto Amorim ML, Oliveira Junior AE, Haddad V (2010). Envenenamento fatal por baiacu (Tetradontidae) – relato de um caso em criança. Rev Soc Bras Med Trop.

[5] Oliveira JS, Pires Junior OR, Morales RAV, Bloch Junior C, Schwartz CA, Freitas JC (2003). Toxicity of puffer fish – two species (Lagocephalus laevigatus, Linaeus 1766 and Sphoeroides spengleri, Bloch 1785) from the Southeastern Brazilian coast. J Venom Anim Toxins incl Trop Dis.

